# Individual and combined effects of ischemic conditioning strategies on infarct size after myocardial ischemia reperfusion

**DOI:** 10.1038/s41598-025-17442-y

**Published:** 2025-08-30

**Authors:** Ahmed Elmahdy, Maryna Krasnikova, Abhishek Jha, Tetiana Pylova, Aaron Shekka Espinosa, Ermir Zulfaj, Erik Axel Andersson, Mana Kalani, Aditi Banerjee, Ola Hammarsten, Elmir Omerovic, Björn Redfors

**Affiliations:** 1 Department of Molecular and Clinical Medicine, Institute of Medicine, University of Gothenburg, Blå stråket 5 B Wallenbergslab/SU, 413 45 Gothenburg, Sweden; 2 Wallenberg Centre for Molecular and Translational Medicine, Institute of Medicine, University of Gothenburg, Gothenburg, Sweden; 3 Department of Clinical Chemistry, Sahlgrenska University Hospital, Region Västra Götaland, Gothenburg, Sweden; 4 Department of Cardiology, Sahlgrenska University Hospital, Gothenburg, Sweden

**Keywords:** Ischemic preconditioning, Ischemic remote perconditioning, Ischemic postconditioning, Myocardial infarction, Troponin I, Cardiology, Experimental models of disease, Preclinical research

## Abstract

Ischemic preconditioning (PreC), remote perconditioning (PerC), and postconditioning (PostC) are known to reduce myocardial infarct size, but their relative efficacy and potential additive effects remain unclear. This study compared the individual and combined effects of PreC, PerC, and PostC on infarct size and cardiac troponin I (cTnI) levels in a rat model of myocardial ischemia–reperfusion. Fifty-four male Sprague–Dawley rats underwent 40 min of coronary occlusion followed by 2 h of reperfusion. They were randomized into six groups: Control, PreC, PerC, PostC, PerC + PostC, or PreC + PerC + PostC. Infarct size was measured using Evans blue/TTC staining, and cTnI levels were assessed. All conditioning strategies significantly reduced infarct size and cTnI levels compared to control (*p* < 0.001). PreC and PreC + PerC + PostC were the most effective, while PostC was the least. No additive benefit was seen when combining PreC with other strategies (*p* = 0.9) or PerC with PostC (*p* = 0.9). These findings suggest that PreC provides the greatest cardioprotection, and combining conditioning strategies does not enhance outcomes, possibly due to overlapping protective mechanisms.

## Introduction

Ischemic conditioning is a promising cardioprotective strategy aimed at mitigating ischemia–reperfusion injury by inducing brief, non-lethal ischemic episodes that enhance tissue resistance to subsequent prolonged ischemia^1–3^. Based on the timing relative to the index ischemic event, ischemic conditioning is categorized into three main types: preconditioning (PreC) before ischemia, perconditioning (PerC) during ischemia, and postconditioning (PostC) at the onset of reperfusion^1,3^.

Among these, ischemic preconditioning—first described in a canine model in 1986—remains the most extensively studied and consistently effective strategy^4,5^. It significantly reduces infarct size across various animal models, including rodents, pigs, and primates^5,6^. However, its reliance on pre-ischemia application limits clinical feasibility in acute unpredictable settings.

To overcome this limitation, alternative strategies applicable during or after ischemia—such as remote perconditioning and ischemic postconditioning—have been explored^7–11^. While preclinical studies, particularly in large animals, support their cardioprotective potential, results in rodent models and clinical trials have been inconsistent, with variable effects on infarct size and myocardial salvage^3,10–20^.

Given the variability, combining PerC and PostC— the two strategies applicable in acute settings—has been proposed to enhance cardioprotection. However, findings remain inconsistent, highlighting the need for further investigation^10,11^.

In this study, we systematically evaluate the cardioprotective efficacy of PreC, PerC, and PostC—both individually and in combination—in a rat model of ischemia–reperfusion injury by assessing their impact on infarct size and plasma troponin I levels^21^.

## Methods and materials

### Rats

Fifty-four male Sprague–Dawley rats (6–8 weeks old, 250–350 g) were obtained from Janvier Labs (Le Genest-Saint-Isle, France). The rats were acclimatized for one week at the Laboratory for Experimental Biomedicine (Gothenburg, Sweden) prior to the experiment. They were housed in standard cages under controlled conditions (21 °C, 12-h light/dark cycle) with ad libitum access to standard laboratory chow and water. All procedures were conducted in compliance with institutional and national guidelines for animal research and were approved by the Gothenburg Animal Ethics Committee (Dnr 5.8.18–11,014/2023) and performed in accordance with ARRIVE guidelines.

### Ischemia–reperfusion model

Surgeries was performed as previously described^2^. Anesthesia was induced with an intraperitoneal injection of ketamine (120 mg/kg) and xylazine (5 mg/kg). To maintain anesthesia, an intravenous infusion of ketamine (0.125 mg/mL) and xylazine (3 mg/mL) in Ringer’s solution was administered via a lateral tail vein cannula and continued until euthanasia following the 2-h reperfusion period. End-tidal CO₂ levels were continuously monitored using CapStar-100 (CWE Inc.) and maintained between 5–6%. Additionally, body temperature, heart rate, oxygen saturation, and respiratory parameters were continuously recorded throughout the procedure.

A left thoracotomy was performed through the fourth intercostal space to access the heart. The left anterior descending (LAD) artery was ligated 3–4 mm distal to its origin using a 6.0 silk suture (Ethicon Inc., NJ, USA). LAD occlusion was confirmed by (i) left ventricular blanching, (ii) ECG changes, and (iii) regional akinesia on echocardiography. Reperfusion was initiated by loosening the suture.

Remote ischemic perconditioning was induced by temporary ligation of the left femoral artery with a 6.0 silk suture. This consisted of four cycles of 5-min occlusion followed by 5-min reperfusion. Effective occlusion was visually confirmed by the absence of distal blood flow.

### Study design

**Fig. 1:**
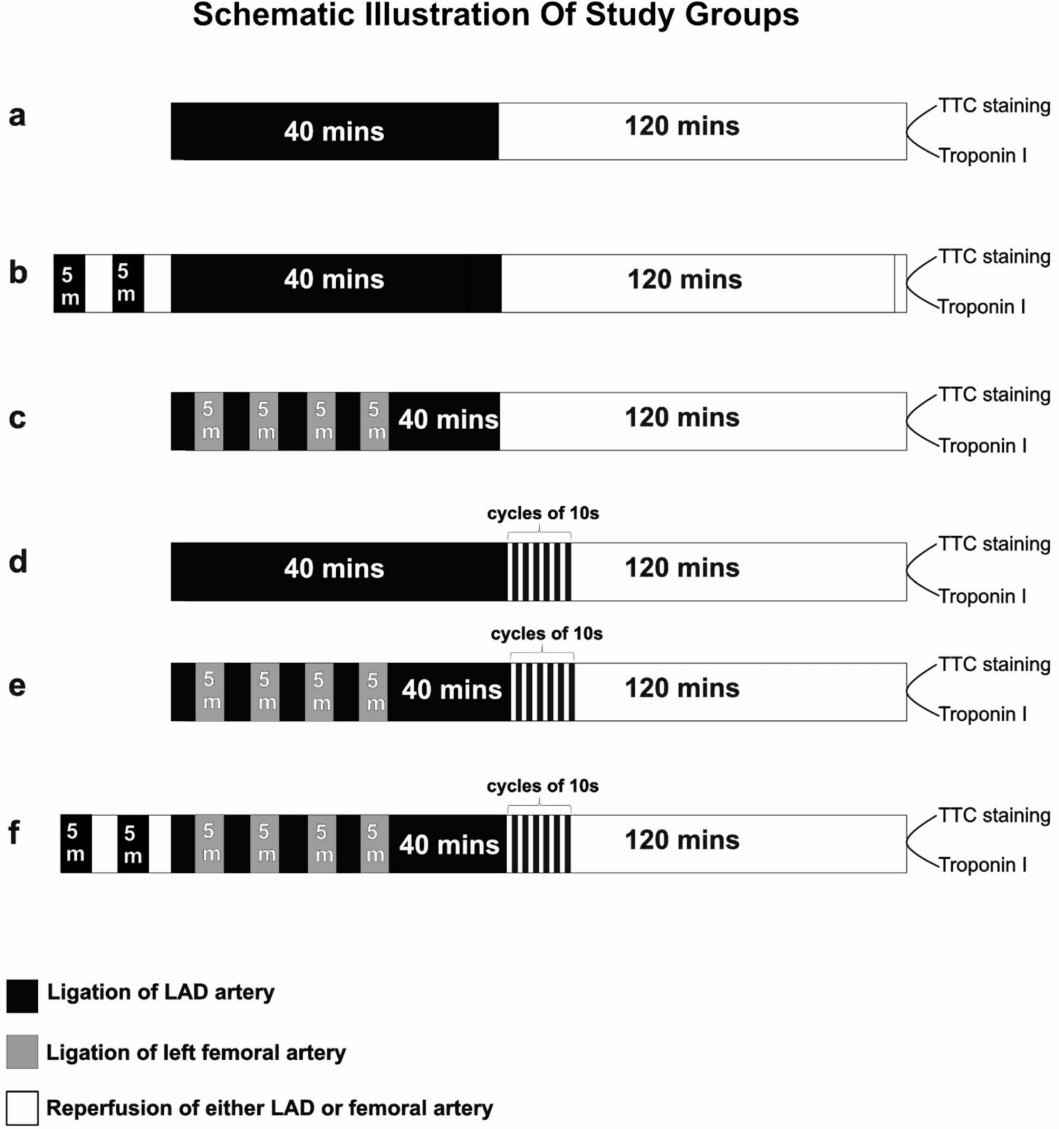
Experimental protocols for ischemia–reperfusion and ischemic conditioning interventions. All groups underwent 40 min of left anterior descending artery (LAD) occlusion followed by 120 min of reperfusion. (**A**) Control group: LAD occlusion and reperfusion without any ischemic conditioning. (**B**) Ischemic preconditioning (PreC): Two cycles of 5 min LAD occlusion/reperfusion before ischemia. (**C**) Remote ischemic perconditioning (PerC): Four cycles of 5 min femoral occlusion/reperfusion during ischemia. (**D**) Ischemic postconditioning (PostC): Six cycles of 10 s LAD occlusion/reperfusion at reperfusion onset. (**E**) PerC + PostC: Combination of PerC and PostC. (**F**) PreC + PerC + PostC: Combination of all three conditioning strategies. Infarct size was assessed using TTC staining, and myocardial injury was evaluated by troponin I measurement. Black bars represent LAD occlusion, gray bars femoral artery occlusion, and white bars reperfusion.

This study evaluated the cardioprotective effects of pre-, post- and per-conditioning on myocardial infarction in a rat model. All rats underwent 40 min-index ischemia followed by 2 h of reperfusion. In addition, rats were randomized to the three types of conditioning as well as to combinations of the conditioning types. Specifically, fifty-four rats were randomly assigned to the following six experimental groups (n = 9 per group): (A) **Control group:** No conditioning intervention; (B) **PreC:** Two cycles of 5-min LAD ischemia and 5-min reperfusion were applied before index ischemia; (C) **PerC:** Four cycles of left femoral artery occlusion (5 min) and reperfusion (5 min) during the index ischemia; (D) **PostC:** Six cycles of 10-s LAD ischemia and 10-s reperfusion were initiated immediately after the index ischemia; (E) **PerC + PostC:** Both PerC and PostC; (F) **PreC + PerC + PostC:** A combination of all three ischemic conditioning strategies. A schematic representation of the experimental protocol is provided in Fig. 1**.**

### Infarct size assessment

Myocardial infarct size was determined using Evans blue and 2,3,5-triphenyltetrazolium chloride (TTC) double staining, as previously described^2^. At the end of the 2-h reperfusion period, the LAD was re-occluded, and 5% Evans blue dye was administered via the lateral tail vein to demarcate non-ischemic myocardium. Hearts were rapidly excised, rinsed in saline, and transversely sectioned into five 2-mm-thick slices from apex to base. Slices were incubated in 1% TTC solution at 37 °C for 10 min in the dark to distinguish viable (red) from infarcted (white) myocardium, then fixed in 10% neutral-buffered formalin and immersed in phosphate buffer (pH 7.4) for 10 min. Digital images of each slice were acquired using a flatbed scanner (Seiko Epson, Japan). Image analysis was performed using ImageJ (v1.34, NIH, USA) to quantify infarcted, viable, and non-ischemic regions. Infarct size was expressed as a percentage of the area at risk , excluding the Evans blue–stained non-ischemic zones. All measurements were performed by two independent, blinded investigators. In cases of discrepancy, consensus was reached through evaluation by a third blinded reviewer.

### Troponin I analysis

Plasma samples were stored at -80 °C until analysis. Cardiac troponin I (cTnI) levels were determined using the Abbott Alinity assay, with a LoB of 1 ng/L, an LoQ of 5.1 ng/L, an analytical CV of 10%, and a 99th percentile reference value of 26 ng/L.

### Exclusion criteria

Animals were excluded based on predefined criteria: (1) failure to position the LAD suture within two attempts; (2) excessive post-occlusion bleeding (≥ 3-soaked cotton swabs); (3) sustained heart rate < 250 bpm; (4) sustained end-tidal CO₂ > 7.5%; or (5) incomplete or technically inadequate TTC staining. Group allocation was randomized, and infarct size analysis was performed by an investigator blinded to treatment groups^22^.

### Statistical analysis

Data were analyzed using R software (v4.2.0). All figures were generated using R, with additional formatting and design adjustments completed in Affinity Designer. Normality was tested using the Shapiro–Wilk test. Group comparisons were performed using one-way ANOVA, followed by Tukey’s post hoc test for pairwise comparisons. Results are expressed as mean ± SD, with significance set at *p* < 0.05.

## Results

### Baseline physiological characteristics

Physiological parameters—including age, body weight, temperature, heart rate, end-tidal CO₂, and oxygen saturation—did not differ significantly between experimental groups **(**Table 1**).** All rats survived the surgery and no procedural mortality was observed.

**Table 1:** Physiological characteristics of rats exposed to different types of ischemic conditioning.

Variable	Control	PreC	PerC	PostC	PerC + PostC	PreC + PerC + PostC
Number	9	9	9	9	9	9
Age (weeks)	7.0 ± 0.5	7.0 ± 0.4	7.1 ± 0.4	6.9 ± 0.3	7.2 ± 0.3	7.1 ± 0.4
Weight (g)	320 ± 25	325 ± 20	330 ± 22	322 ± 24	328 ± 19	323 ± 21
Temperature (°C)	37.4 ± 0.4	37.6 ± 0.3	37.5 ± 0.3	37.3 ± 0.2	37.6 ± 0.2	37.5 ± 0.3
Heart rate (Bpm)	256 ± 14	260 ± 12	249 ± 18	265 ± 15	252 ± 17	255 ± 20
ET-CO2 (%)	5.5 ± 0.4	5.3 ± 0.5	5.7 ± 0.3	5.7 ± 0.2	5.3 ± 0.3	5.6 ± 0.4
O2 saturation (%)	98.9 ± 0.3	99.0 ± 0.2	98.9 ± 0.2	98.8 ± 0.2	99.0 ± 0.3	99.0 ± 0.2

Data are shown as mean and standard deviation.

PreC, ischemic preconditioning; PerC, remote ischemic perconditioning; PostC, ischemic postconditioning; ET, end-tidal; BPM, beat per minute.

### Infarct size

**Fig. 2:**
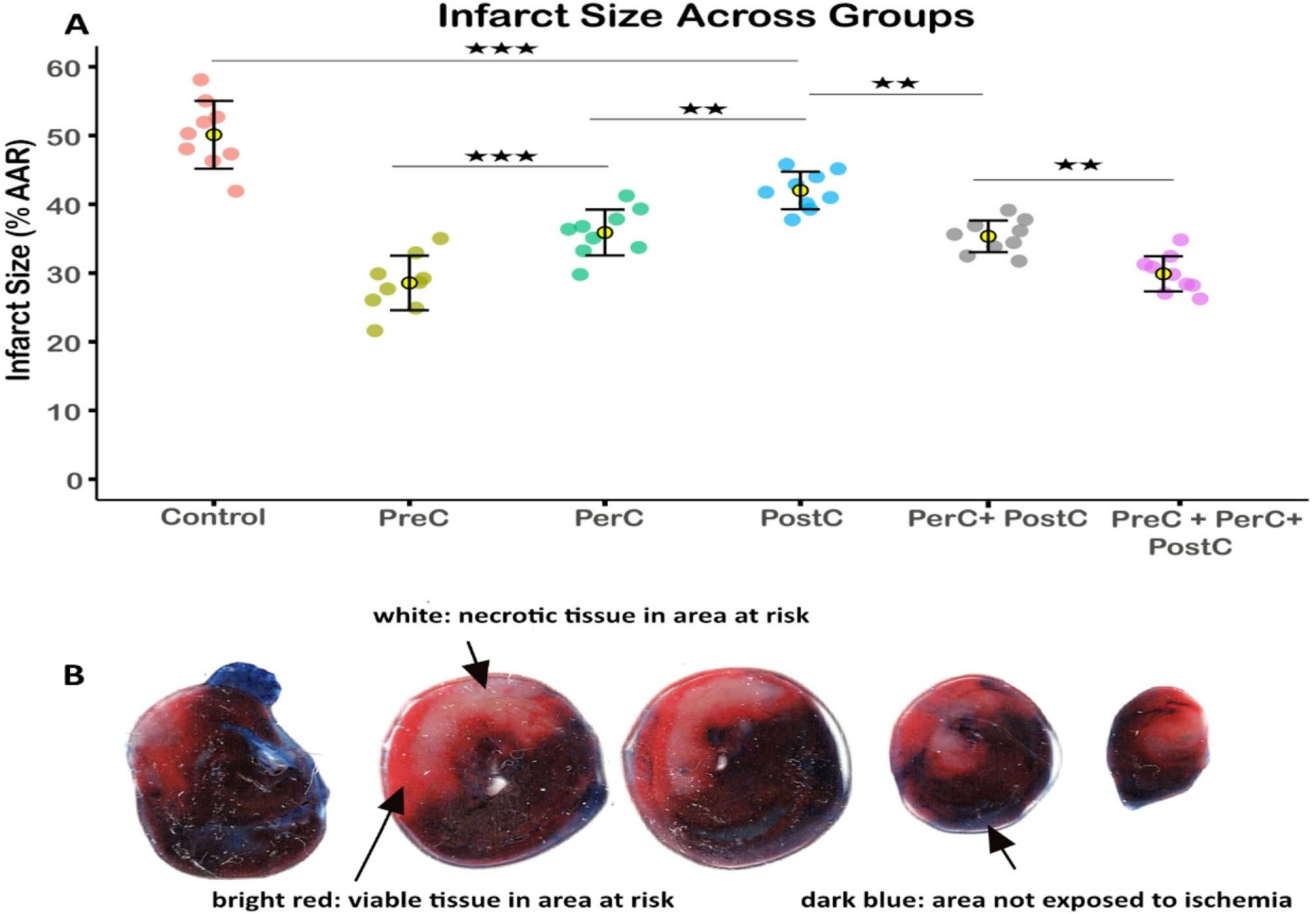
Infarct size across experimental groups. (**A**) Infarct size, expressed as a percentage of the area at risk (AAR), was significantly higher in the control group compared to all other groups (*p* < 0.001). The smallest infarcts were observed in the PreC and PreC + PerC + PostC groups, with no significant difference between them (*p* = 0.9). PerC and PerC + PostC significantly reduced infarct size compared to PostC (*p* < 0.01), but the combination of PerC + PostC did not provide additional benefit over PerC alone (*p* = 0.9). Each dot represents one rat (n = 9 per group). Yellow circles indicate group means, and error bars represent standard deviation. Statistical comparisons were performed using one-way ANOVA followed by Tukey’s post hoc test. ** = *p* < 0.01, *** = *p* < 0.001. (**B**) Representative images of whole hearts stained with triphenyltetrazolium chloride (TTC). Dark blue regions indicate myocardium not subjected to ischemia. The bright red and white areas represent the area at risk (AAR), with bright red indicating viable myocardium and white indicating necrotic tissue.

Infarct size, expressed as a percentage of the area at risk, differed significantly between experimental groups. The control group exhibited the largest infarct size (50.6% ± 4.9%), significantly larger than in all other groups (*p* < 0.001). The smallest infarcts were observed in the PreC group (28.6% ± 3.9%) and the PreC + PerC + PostC group (29.4% ± 2.5%). These two groups had statistically significantly smaller infarct size than all other groups with no significant difference between them (*p* = 0.9). Infarct size in the PerC group (35.6% ± 3.3%) was significantly smaller than in the PostC group (42% ± 2.7%, *p* < 0.01). While combined PerC and PostC (36.6% ± 2.3%) conferred greater protection than PostC (*p* < 0.01), it did not differ compared to PerC alone (*p* = 0.9). These findings are illustrated in Fig. 2**.**

### Troponin I levels

**Fig. 3:**
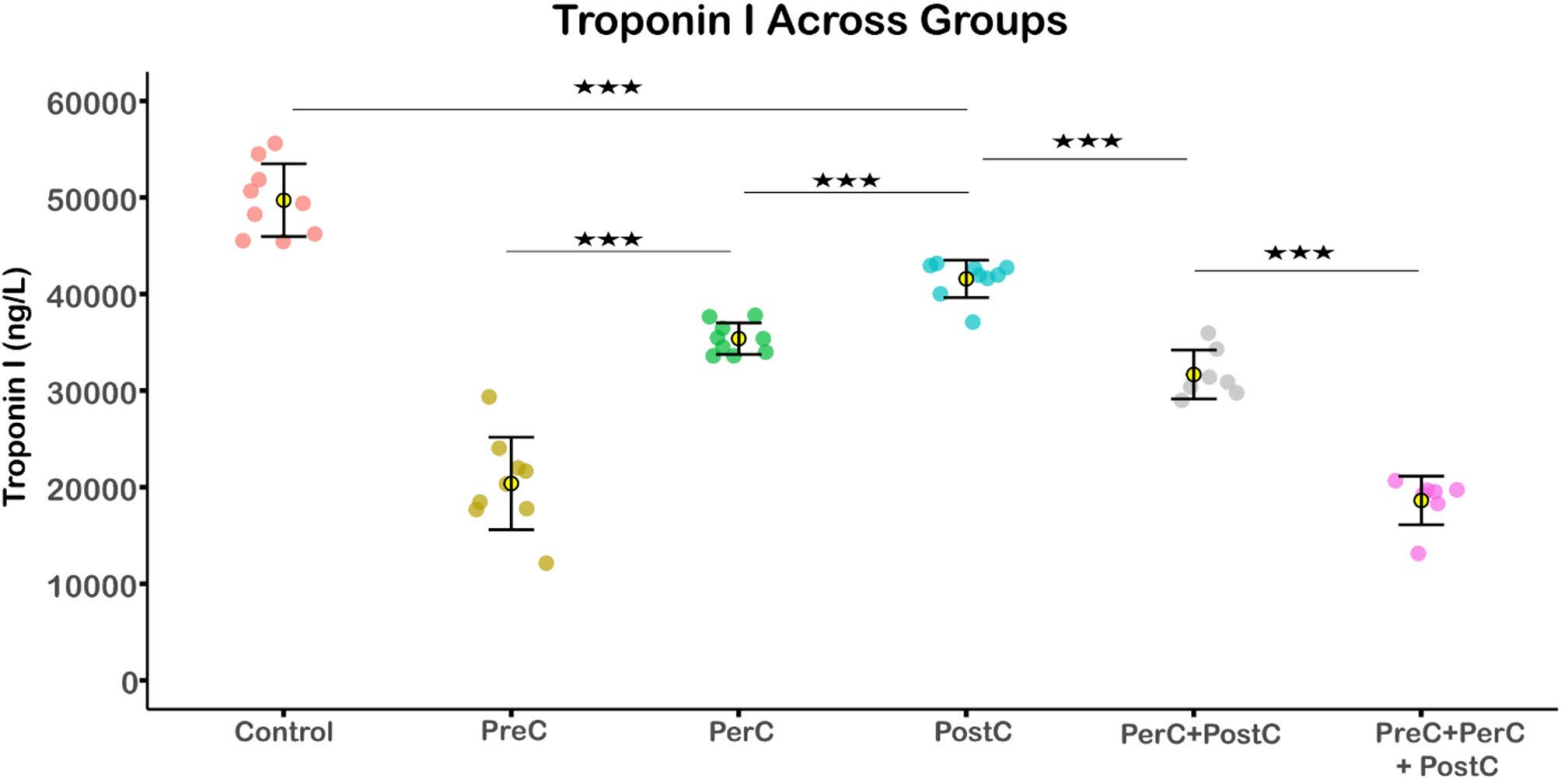
Troponin I levels across experimental groups. Troponin I (cTnI) levels were highest in the control group, and significantly higher than in all other groups (*p* < 0.001). The lowest levels were observed in the PreC and PreC + PerC + PostC groups, with no significant difference between them (*p* = 0.9). PerC and PerC + PostC reduced cTnI levels more than PostC (*p* < 0.001), but the combination of PerC + PostCdid not provide additional benefit over PerC alone (*p* = 0.18). Each dot represents one rat (n = 9 per group). Yellow circles indicate group means, and error bars represent standard deviation. Statistical analysis was performed using one-way ANOVA followed by Tukey’s post hoc test. *** = *p* < 0.001.

Plasma cardiac troponin I (cTnI) levels were highest in the control group (49 723 ± 3 765 ng/L), significantly exceeding all other groups (*p* < 0.001). Similar to the histological infarction size, cTnI levels were significantly lower in the PreC (20 386 ± 4 796 ng/L) and PreC + PerC + PostC (18 625 ± 2 517 ng/L) groups than other groups, with no significant difference between them (*p* = 0.9). Among the other groups, PerC and PerC + PostC resulted in significantly lower cTnI levels than PostC (*p* < 0.001 for both), with no significant difference between PerC alone and PerC + PostC. These findings are shown in Fig. 3**.**

## Discussion

The most important findings of our study is that in a controlled experimental setting (i) PreC, PerC, and PostC all significantly reduce histological infarct size and cTnI release after myocardial ischemia–reperfusion, with the most pronounced reduction in infarct size observed with PreC and the least pronounced effect observed with PostC; and (ii) there were no additive effect of PostC to PerC or PostC and PerC to PreC suggesting a potential ceiling effect when combining conditioning types.

Both PostC and PerC have reduced infarct size in preclinical models, though PostC has shown more variable efficacy^10,11^. This inconsistency may stem from protocol-dependent factors, such as the number, duration, and timing of ischemia–reperfusion cycles. Shorter cycles or delayed initiation have been shown to reduce protective effects^3,10^. Conversely, PerC has demonstrated consistent efficacy in large animal models but remains inconsistent in rodent studies, where species-specific metabolic and vascular differences, as well as protocol variations, may contribute to variability^11,15^.

In clinical settings, the efficacy of PostC and PerC remains inconclusive. While some studies report PostC-induced infarct size reduction in STEMI patients, as measured by Single-Photon Emission Computed Tomography and cardiac biomarkers (CK-MB, troponin I), others show no improvement in myocardial salvage or infarct size^3,12–14^. Similarly, PerC trials have demonstrated reductions in injury markers and improved myocardial salvage, but without significant impact on final infarct size, raising questions about the clinical significance of these improvements^17–19^.

In this study, both PostC and PerC conferred cardioprotection by reducing infarct size compared to the control group. However, PerC proved more effective than PostC, while neither approach matched the superior efficacy of ischemic preconditioning. Troponin I release closely paralleled infarct size reduction across groups. The inconsistencies in both experimental and clinical studies call into question the reliability of PostC and PerC as standalone interventions, prompting research into their combined application for enhanced cardioprotection.

A rat study identified an optimized PerC + PostC protocol—consisting of four cycles of 5-min unilateral limb occlusion (PerC) during ischemia, followed by six cycles of 10-s occlusion-reperfusion (PostC) at reperfusion onset—which yielded maximal infarct size reduction^11^. However, a subsequent study failed to replicate these findings, with the only methodological difference being the use of bilateral rather than unilateral limb occlusion^10^. Clinical trials have shown similarly inconsistent outcomes. The RIPOST-MI trial found that adding PostC to PerC in STEMI patients failed to enhance cardioprotection, as assessed by CK-MB release^19^. In contrast, the LIPSIA trial reported a significantly higher myocardial salvage index (MSI) in the PerC + PostC group compared to conventional PCI, though this did not translate into reduced infarct size or improved long-term outcomes^13^.

In our study, we applied the same unilateral optimized protocol as Xin et al.^11^ but observed no added benefit of combining PerC and PostC over PerC alone. Likewise, triple conditioning (PreC + PerC + PostC) did not further reduce infarct size or cTnI levels over PreC alone, suggesting no cumulative benefit from combining strategies. Although combining PerC and PostC is theoretically appealing, both animal and human studies have failed to demonstrate consistent additive effects.

**Pushing the Protective Ceiling of Ischemic Conditioning.**

One plausible explanation for this ceiling effect is the extensive convergence of cardioprotective signaling cascades^23,24^. IPC, IPost, and RIPerC differ in their temporal windows and initiating mechanisms.

- IPC triggers protection before ischemia through early activation of PKC-ε, Akt, ERK1/2, and STAT3, followed by a delayed transcriptional phase involving JAK–STAT and p38 MAPK signaling^25–27^.
- IPost acts exclusively at reperfusion, rapidly engaging Akt, ERK1/2, eNOS, and STAT3 via autacoid signaling, but without a delayed phase^27–29^.
- RIPerC, initiated during ongoing ischemia, relies on remote neural and humoral inputs, with robust STAT3 phosphorylation often driven by circulating cytokines^30,31^.

**Fig. 4:**
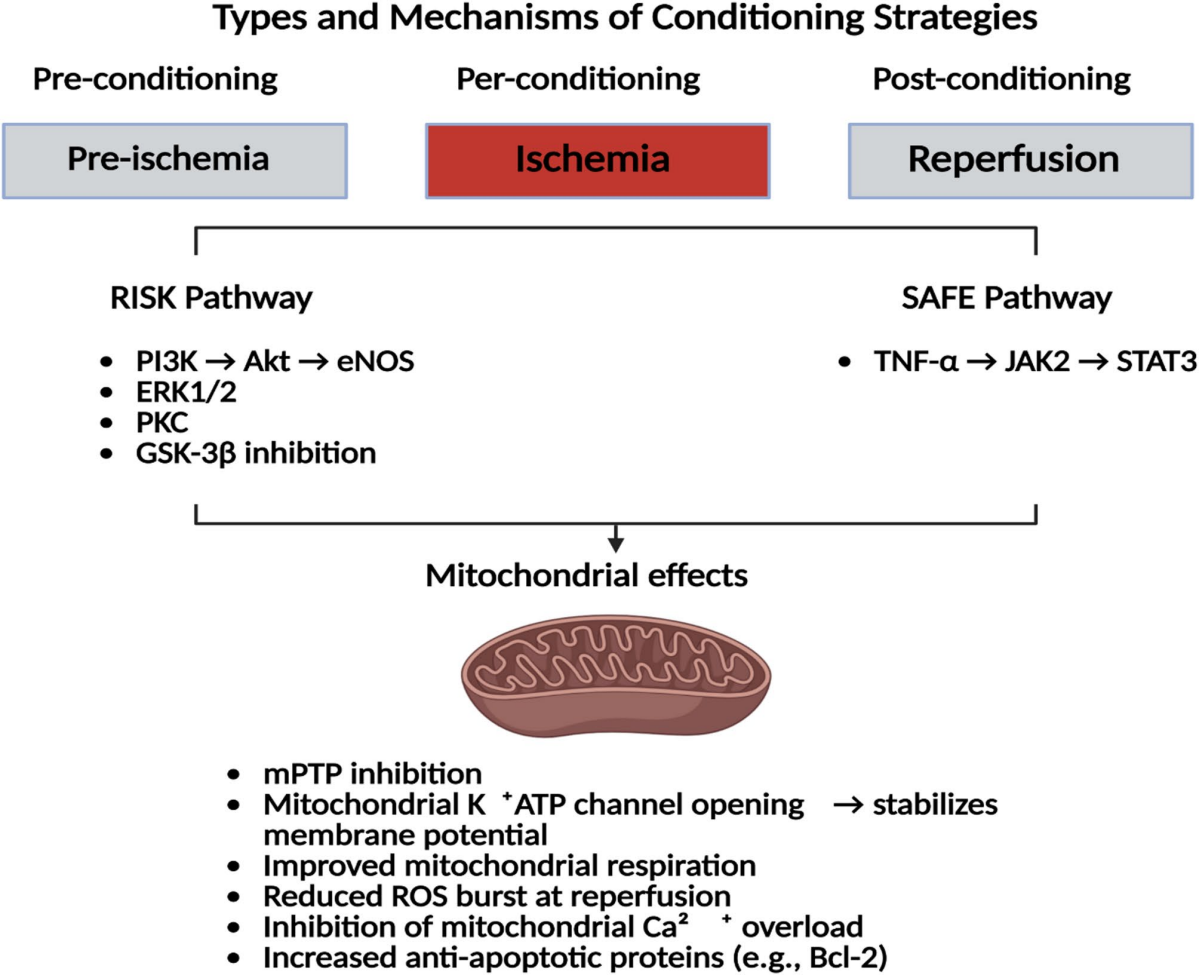
Mechanisms of cardioprotection in ischemic conditioning. Ischemic pre-, per-, and post-conditioning activate pro-survival signaling cascades—primarily the RISK (PI3K–Akt–eNOS, ERK1/2, PKC, GSK-3β) and SAFE (TNF-α–JAK2–STAT3) pathways—which converge on mitochondria to inhibit mPTP opening, stabilize membrane potential, reduce oxidative stress and calcium overload, and enhance anti-apoptotic signaling. *Created by BioRender.*

Despite these upstream differences, all three strategies converge on the RISK (PI3K–Akt, ERK1/2) and SAFE (JAK–STAT3) pathways and downstream effectors including nitric oxide (NO), PKG activation, and inhibition of mitochondrial permeability transition pore (mPTP) opening. This convergence may impose a ceiling effect, beyond which additional stimuli yield no further benefit^27,28^, as illustrated in Fig. 4**.**

The variability observed in both preclinical and clinical studies reflects a broader limitation in the field: the lack of systematic optimization of conditioning algorithms. Most studies, including ours, rely on legacy protocols developed in early proof-of-concept experiments, which—while offering consistency and comparability—may not represent optimal configurations^32,33^. The absence of additive protection in our study may therefore result not only from pathway saturation but also from suboptimal algorithm selection (timing, duration, or cycle number). Rational, head-to-head algorithmic design studies are warranted to systematically map protocol parameters and identify configurations that maximize efficacy.

Further, incomplete engagement of other molecular regulators may contribute to the variability of cardioprotection. Redox signaling plays a dual role: while moderate ROS levels promote pro-survival pathways—PKC and ERK1/2 activation-, sustained oxidative stress can impair signaling fidelity and exacerbate injury^34^. Mitochondrial dysfunction, in turn, activates the NLRP3 inflammasome, driving pyroptotic cell death. Targeting mitochondrial ROS or NLRP3 pharmacologically has been proposed as a way to reinforce cardioprotection^35,36^*.* Moreover, mitophagy, via PINK1/Parkin or BNIP3-dependent pathways, maintains mitochondrial integrity and restrains inflammation; impaired mitophagy, particularly in the context of metabolic syndrome, may blunt the efficacy of conditioning. Interventions that fine-tune mitophagic flux may further enhance the efficacy of ischemic conditioning^37^*.*

Together, these insights support a multidimensional optimization framework: future studies should (1) explore the combinatorial space of conditioning parameters and (2) integrate targeted modulation of redox homeostasis, mitochondrial quality control, and inflammasome signaling. This integrative strategy may help advance ischemic conditioning toward more robust, reproducible, and clinically translatable cardioprotection.

## Conclusion

Our study demonstrated that PreC, PerC, and PostC each independently reduced infarct size and troponin I release, with PreC providing the most pronounced reductions. Neither combining PerC and PostC nor combining PreC, PerC, and PostC conferred additional cardioprotection, suggesting a ceiling effect due to overlapping mechanism.

## Limitations

Only male Sprague–Dawley rats were used, which may limit the generalizability of the findings to female subjects. Given that sex differences in ischemic tolerance and cardioprotection are well documented^38^, future studies should investigate sex-specific responses to ischemic conditioning, as hormonal and metabolic differences may influence cardioprotective efficacy.

This study focused on acute outcomes—namely infarct size and troponin I levels—which, although established markers of myocardial injury, do not capture long-term functional consequences. Future research should incorporate echocardiographic evaluations to determine parameters such as left ventricular remodeling and systolic function. Invasive hemodynamic measurements were not performed. Future studies should incorporate such assessments to further elucidate the functional consequences of different conditioning strategies.

This study was conducted in healthy rats, appropriate for controlled mechanistic evaluation as outlined in the IMPACT criteria^39^, but not reflective of the clinical profile of myocardial infarction patients. It is well established that comorbidities such as diabetes, aging, and hyperlipidemia, and commonly used medications including opioids, antiplatelets, and anesthetics can attenuate or abolish cardioprotective signaling^40–43^. Incorporating these confounders into future preclinical models is essential to assess the true translational potential of candidate cardioprotective strategies.

## Data Availability

The datasets generated during and analysed during the current study are available from the corresponding author on reasonable request.

## Abbreviations

PreC: Ischemic preconditioning
PerC: Remote ischemic perconditioning
PostC: Ischemic postconditioning
LAD: Left anterior descending artery
cTnI: Cardiac troponin I
TTC: 2,3,5-Triphenyltetrazolium chloride
